# Middle-aged and older people with urgent, unaware, and unmet mental health care needs: Practitioners’ viewpoints from outside the formal mental health care system

**DOI:** 10.1186/s12913-022-08838-x

**Published:** 2022-11-23

**Authors:** Akinori Takase, Yuki Matoba, Tsutomu Taga, Kae Ito, Tsuyoshi Okamura

**Affiliations:** 1 Department of Public Policy, Faculty of Socio-Symbiosis, Taisho University, Toshima, Tokyo, Japan; 2 Hurusato No Kai, Taito, Tokyo, Japan; 3 Research Team for Promoting Independence and Mental Health, Tokyo Metropolitan Institute of Gerontology, Itabashi, Tokyo, Japan; 4 Research Team for Human Care, Tokyo Metropolitan Institute of Gerontology, Itabashi, Tokyo, Japan

**Keywords:** Mental health services, Universal health coverage, Unmet health care needs

## Abstract

**Background:**

Mental health challenges are highly significant among older individuals. However, the non-utilization of mental health services increases with age. Although universal health coverage (UHC) was reported to reduce unmet health care needs, it might not be sufficient to reduce unmet mental health care needs from a clinical perspective. Despite the existence of UHC in Japan, this study aimed to explore the factors related to the non-utilization of formal mental health care systems among middle-aged and older people with urgent, unaware, and unmet mental health care needs.

**Methods:**

Purposeful sampling was used as the sampling method in this study by combining snowball sampling and a specific criterion. The interviewees were nine practitioners from four sectors outside the mental health care system, including long-term care, the public and private sector, as well as general hospitals in one area of Tokyo, where we had conducted community-based participatory research for five years.

The interviews were conducted by an interdisciplinary team, which comprised a psychiatrist, a public health nurse from a non-profit organization, and a Buddhist priest as well as a social researcher to cover the broader unmet health care needs, such as physical, psychosocial, and spiritual needs. The basic characteristics of the interviewees were enquired, followed by whether the interviewees had case of middle-aged or older individuals with urgent, unaware, and unmet mental health care needs. If the answer was yes, we asked the interviewees to describe the details. The interviews pertinent to this study were conducted between October 2021 and November 2021.

In this study, we adopted a qualitative descriptive approach. First, we created a summary of each case. Next, we explored the factors related to the non-utilization of formal mental health care systems by conducting a thematic analysis to identify the themes in the data collected.

**Results:**

The over-arching category involving “the factors related to an individual person” included two categories, as follows: 1) “Individual intrinsic factors,” which comprised two sub-categories, including “difficulty in seeking help” and “delusional disorders,” and 2) “family factors,” which comprised “discord between family members,” “denial of service engagement,” “multiple cases in one family,” and “families’ difficulty in seeking help.”

The over-arching category “the factors related to the systems” included four categories, as follows: 1) “Physical health system-related factors,” which comprised “the indifference of physical healthcare providers regarding mental health” and “the discontinuation of physical health conditions,” 2) “mental health system-related factors,” which comprised “irresponsive mental health care systems” and “uncomfortable experiences in previous visits to clinics,” and 3) “social service system-related factors,” which comprised “the lack of time to provide care,” “social service not allowed without diagnosis,” and “no appropriate service in the community,” as well as 4) “ the lack of integration between the systems.”

Apart from the aforementioned factors, “the community people-related factor” and “factors related to inter-regional movements” also emerged in this study.

**Conclusions:**

The results of this study suggest a specific intervention target, and they provide further directions for research and policy implementation. The suggested solutions to the issues pertinent to this study are as follows: the recognition of the ways in which older people may inadequately understand their health or be unaware of available services, the building of a therapeutic alliance for “the individual intrinsic factors.” Regarding the “family factors,” the solutions include the provision of particularly intensive care for families with family discords, families with multiple cases, and families who find it difficult to seek help, as well as making intensive efforts for ensuring early involvement after contact with health care services. Regarding the “the factors related to the systems,” the solutions include the implementation of mental health education campaigns aimed at enhancing mental health knowledge among non-mental health professionals, as well as formulating and implementing reforms ensuring that such professionals are increasingly responsible especially with regard to emergency inpatient care. It also include listening without ageism in clinical practice, the expansion of social services regarding human resources and the flexibility of use which increases the breadth of the types of care, as well as facilitating the integration between the associated health care systems. Further suggestions include encouraging community residents to join social security systems as well as the provision of particularly intensive care for people who have just moved in.

## Introduction

The Japanese society continues to age rapidly. In 2022, the proportion of people aged 65 years and above was 29.1%, which is the highest worldwide. By the year 2050, this number is estimated to increase to approximately 40% [1]. Because dementia is often associated with psychiatric symptoms [2] and disorders [3], differential diagnosis remains a challenge among clinicians. However, no studies on the prevalence of psychiatric disorders among the older Japanese population have been conducted, thereby suggesting methodological difficulties and/or low public concern.

Mental health challenges are significant among older individuals. In 1999, Jeste et al. presented a consensus statement [4] in which they predicted that 20% of older individuals would experience mental health challenges. Furthermore, according to Age Concern England [5], 6% of older people in the UK are predicted to experience mental health challenges. Moreover, in 2015, a large epidemiological study conducted in the USA [6] reported that 11.4%, 6.8%, 3.8%, and 14.5% of older individuals experienced anxiety, depressive, addiction, and personality disorders, respectively, in the past year. Similarly, in 2017, a large study conducted in Europe [7] reported that 23% of older individuals had mental disorders.

Specific models of care have been proposed and implemented throughout the society to address mental health challenges among older people [8]. For affective disorders, the collaborative care model is reported to be feasible and significantly more effective than the usual care model [9]. This model comprises four components: 1) a multi-professional approach to patient care, 2) a structured management plan tailored to the individual needs of the patient, 3) proactive follow-up aimed at delivering evidence-based treatments, and 4) processes for enhancing inter-professional communication, such as routine and regular team meetings and/or shared records [10]. Additionally, older persons suffering from schizophrenia have higher frequency and severity levels of physical diseases, and yet, they receive significantly less than adequate health care [11]. The combination of psychosocial-skill training with preventive healthcare intervention is reported to have positive effects [12].

Generally, broad or universal health coverage is an effective strategy for reducing unmet medical needs. According to an analysis conducted by the National Health Service in China, universal health coverage (UHC) reduced the levels of unmet healthcare needs [13]. As reported by the World Health Organization [14], through economic development, many countries are moving towards UHC.

Despite the societal implementations mentioned above, the non-utilization of health care among older people has been reported. The non-utilization of psychotherapy was reported to increase with age in the USA [15]. In Japan, according to Yoshioka [16], the staff of comprehensive community support centers set in every junior high school district to support community-dwelling older people requiring long-term care, were burned out mainly as a result of older people facing mental health challenges. According to a report on community-based participatory research [17], older people who came for free local consultation without medical insurance had increasingly unmet and unaware mental health care needs instead of pure medical needs. Although it is not sufficient, UHC might be a necessary approach for reducing unmet mental health care needs from a clinical perspective.

### Japanese context

Japan’s healthcare system has been changing in response to rapid societal aging. In 1973, UHC, which was established in 1961, became completely free of charge for older people. However, the financial burden was significant, and in 2006, a flat rate of 10% co-payment was introduced for individuals aged 75 years and above [18]. Currently, the co-payment rates for healthcare costs incurred by older individuals range between 0 and 30% depending on income [19]. Furthermore, there is also a co-payment cap, which is also determined based on income. Free access is still maintained, and one can see any specialist at any time.

In 2000, the implementation of the Long-Term Care Insurance Act enabled people aged 65 years and above to use long-term care insurance, with a 10% co-payment. In 2005, owing to the increased number of people living alone and individuals suffering from dementia, community general support centers (CGSC) were set up, where older people could go for advice [20].

In response to the public concern regarding dementia, in 2008, the Japanese Ministry of Health, Labor and Welfare established Medical Centers for Dementia (MCD) to deliver specialized medical services throughout the community [21]. According to a national report, 42% of MCDs were within general hospitals [22], and their advantages were as follows: 1) collaborative assessment between neurology, geriatrics, and psychiatry, 2) the availability of advanced imaging tools, such as magnetic resonance imaging, single photon emission computed tomography, and positron emission tomography, as well as 3) the ability for ensuring inpatient care for people suffering from dementia and multimorbidity.

### Aim

The aim of this study was to explore the factors related to the non-utilization of formal mental health care systems for middle-aged and older people with urgent, unaware, and unmet mental health care needs, despite the existence of UHC. The results may contribute to policy-making in countries on their way toward UHC.

## Methods

### Sample

**Fig. 1:**
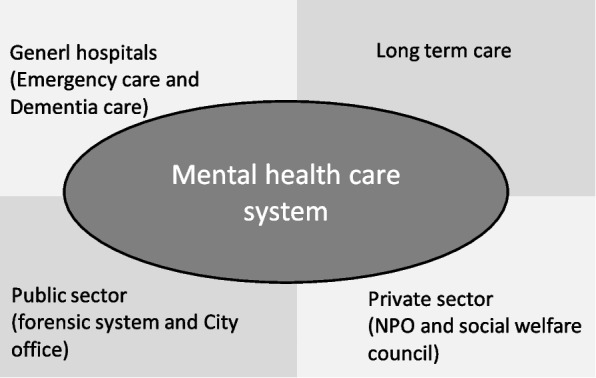
Sample of this study from fields outside the mental health care system

The purposeful sampling method, as described by Palinlkas [23], enabled us to secure information and ensure participants’ availability and willingness to participate as well as their ability to communicate their experiences and opinions. We combined snowball sampling and a specific criterion. Therefore, we chose one area comprising the Itabashi and Toshima wards located at the north-west of the Tokyo metropolitan area, where we had a close relationship with the stakeholders and knew the actual situation because we had previously conducted community-based participatory research in this area for five years [24]. We selected people from organizations outside the mental health care system, who supported middle-aged and older people with urgent, unaware, and unmet mental health care needs. The reasons for this selection were as follows: (a) Previous literature shows that older people who came to institutions outside the mental health care system had increasingly unmet mental health care needs, and they were unaware of their mental health care needs [17], (b) their experiences are not visible because formal mental health care systems have no approaches for gathering and reacting to such outside voices. The participants were selected from four sectors: 1) long term-care, 2) the public sector (forensic system and city office), 3) the private sector (non-profit organizations (NPOs) and social welfare council), as well as 4) general hospitals (Fig. 1). In summary, the inclusion criteria were as follows: (1) persons recruited through snowball sampling, (2) persons that could communicate their experiences and opinions, (3) persons working in the catchment area, and (4) persons working for organizations outside the mental health care system.

We defined the components of formal “mental health care system” as clinics and hospitals specialized for patients suffering from mental illnesses. In Japan, the primary health care system is in its infancy, and every physician regards themselves as a specific medical specialist, such as a psychiatrist or urologist, among other specialties. Accordingly, we could not find appropriate primary health care physicians.

The characteristics of the interviewees are illustrated in Table 1. Regarding the long-term care sector, three persons from three CGSCs participated in the interviews. Regarding the public sector, a certified psychiatrist working for the City Office and a mental health social worker working for the association of mental health social workers participated in the interviews. Regarding the private sector, a staff member of the social welfare council, who manages the volunteer center, and a staff member of the homeless support NPO that provides free and low-cost accommodation for people on welfare payment with nowhere to live participated in this study. Finally, for the general hospitals, two social workers from the two backbone hospitals located in the area participated in the interviews.

**Table 1:** Characteristics of the interviewees

Field	ID	Organization	Certification (in the order in which they are mentioned)	Gender	Age	Brief description of the organization	Interview type
Long-term care	1	CGSC	Certified social worker, Mental health social worker, Care Manager	F	60’s	This CGSC was located in one of the large housing complexes. It received 500–600 consultations a month from individuals and their families, of which approximately 10 cases were ongoing. There were eight staff members	Face to face
	2	CGSC	Certified social worker, Care Manager	M	50’s	This CGSC was located on the ground floor of a large geriatric institution. The interview was accompanied by one staff member, whose certification was a care manager, and who was a female in her 40’s	Face to face
	3	CGSC	Certified social worker, Mental health social worker, Care Manager	F	40’s	This CGSC was located on the ground floor of a geriatric institution. There were six staff members. 60% of consultations involved long-term care insurance, and the rest involved medical care and rights protection	Face to face
Public sector	4	City Office	Certified psychiatrist	F	50’s	She was a part-time psychiatrist who consulted city officers on one case per hour. The subject person was met only once at the health center or at home, accompanied by the city officers	Online
	5	Association of mental health social worker	Mental health social worker	M	50’s	If a person who had been arrested and charged was likely to have a mental health challenge, the lawyer would ask the association to formulate a support plan	Online
Private	6	Social welfare council	Teacher’s license	M	40’s	It supported local residents by supporting the activities of volunteer groups, civic organizations, and non-profit organizations	Face to face
	7	Homeless support (NPO)	Teacher’s license, Social work officer	M	30’s	It ran a free and low-cost accommodation for people on welfare payment that had nowhere to live and assisted with flat transfers as well as employment. There were 22 rooms, with approximately 30 new users every year	Online
General Hospital	8	General hospital (not MCD)	Medical social worker	F	20’s	It was the consulting office of the regional core general hospital (approximately 300 beds), and it received requests from doctors and nurses. Each staff member was responsible for approximately 50 inpatients and outpatients at any time. It had eight staff members	Face to face
	9	General hospital (MCD)	Mental health social worker, Certified social worker	F	40’s	It was the consulting office of the regional core hospital (550 beds), which was certified as an MCD. In addition to consultation, they promoted regional cooperation and provided training as an MCD. It had six staff members	Online

*Note: CGSC* Community general support centers, *MCD* Medical centers for dementia, *NPO* Non-profit organization

All the interviewees had cases of middle-aged and older people with urgent, unaware, and unmet mental health care needs. Because such cases were not originally assumed in their professions, they regarded such cases as essential, and they were willing to participate in discussions regarding such cases.

### Interviews

**Fig. 2:**
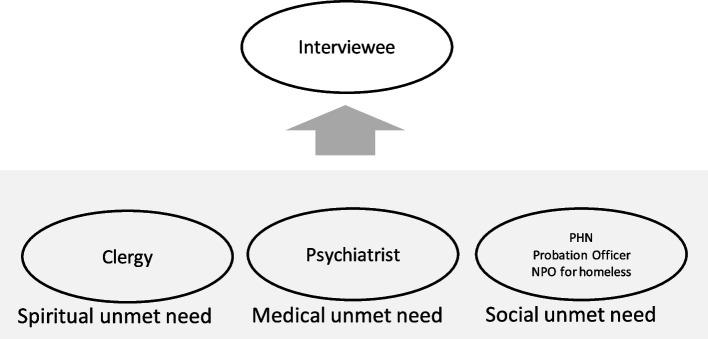
Formation of an interdisciplinary team to cover the comprehensive needs of older people

The interviews were conducted between October 2021 and November 2021 in the private rooms of the interviewees’ offices (five interviews) or through an online conference system upon request (four interviews). Because extant literature on this issue is scarce, the methods were developed from our clinical practice based on a realistic viewpoint. The first author, a psychiatrist, worked with Buddhist priests and homeless support groups to assist in cases that are difficult to support using the conventional health care system. Although this collaboration was driven by clinical necessity, our collaboration was backed by literature. A previous study suggested that the unmet care needs of older people were classified into physical, psychosocial, and spiritual needs [25]. We decided to conduct interviews via an interdisciplinary team comprising a psychiatrist (TO, male, 40’s, working as the director of the public medical research institute, and also working as a clinician for 1–2 days a week), a public health nurse from a non-profit organization supporting homeless individuals (MY, female, 40’s, working as the board member of a support group for homeless individuals while directing the health issues of the users), and a Buddhist priest as well as social researcher (TA, male, 30’s, a Buddhist priest belonging to the Jodo denomination, which is one of the main seven denominations in Japanese Buddhism and is also a university lecturer) to cover the comprehensive needs of older people, as well as their physical, psychosocial, and spiritual needs (Fig. 2).

Basic characteristics, such as age and certifications, were enquired, followed by whether the interviewees had cases of middle-aged and older people with urgent, unaware, and unmet mental health care needs. If the answer was yes, we asked them to describe the details.

We asked the interviewees to talk freely and avoid blocking out information. All the diagnoses were enquired throughout the course of the interviews. Therefore, they were of a wide variety, including those in formal statistical manuals for addressing simple behavioral conditions. We did not confirm whether the diagnoses were made by medical professionals or specialists. In other words, the diagnoses depended on the interviewees’ stories. We included middle-aged people as the topic of the interview. This was because 1) middle-aged people and older people were in continuity, and 2) the interviewees supported middle-aged people around older people.

The interviews were conducted in private rooms or online to avoid being overheard by third parties. All the proper names were initialed when the verbatim transcripts were digitized. The interviewees were assigned an ID number, and a separate comparison chart was maintained.　Written informed consent was obtained from all the participants prior to the interviews.

### Analysis

All the interviews were conducted in Japanese and audio recorded, after which they were transcribed verbatim. Field notes were also prepared during the interviews. We adopted a qualitative descriptive approach [26]. Qualitative description was useful in previous exploratory studies with limited knowledge regarding the topic. The interviews conducted in this study were guided by case presentations because the interviewees were practitioners. Accordingly, the analysis of the data collected comprised two steps. First, we created a summary of each case. Next, we explored the factors related to the non-utilization of formal mental health care systems among older people with urgent, unaware, and unmet mental health care needs. Following the principles of qualitative description methodologies, we conducted a thematic analysis to identify the themes in the data and establish meaningful categories. We allowed the interviewees to talk beyond a specific case, i.e., their general opinions. This information was also considered valuable data. The text fragments were coded using MAXQDA 2018®. Constant comparisons were conducted, and all the authors had access to all the data. Additionally, everything concerning the study was decided in research meetings. All the analyses were conducted in Japanese. After the manuscript was prepared in Japanese, it was translated into English by the authors and a native English-speaker. All the authors agreed that the English version retained the meaning of the analyses.

## Results

### Cases discussed

A summary of the 32 cases involved in this study is presented in Table 2. The number of cases discussed by each interviewee was two to six, with an average of 3.6. The 32 cases included one couple and one pair of sisters, i.e., 34 persons (16 females and 18 males). Concerning age, the number of cases per age group were seven (40’s), three (50’s), four (60’s), eight (70’s), and 10 (80’s), and the remaining two were just “old” sisters. The suspected diagnoses showed wide diversity, and the most frequent category was “unspecified,” (seven cases) followed by “schizophrenia,” (six cases) “dementia,” (five cases), “suicide attempts” (three cases), and “depression” (two cases), which overlapped.

**Table 2:** Summary of the cases

Case	Interviewee	Gender	Age	Suspected diagnosis or condition	Episodes before	Episodes after	Service usage outcome
1	1	Couple	80’s	Dementia	They just moved from a different area. Because whether they have dementia was not clear, the support required was not clear	CGSC suggested seeing a professional doctor, and it succeeded	Clinic
2	1	M	80’s	Delusional disorder	He fought back against the undetectable “noise” from next door	CGSC suggested seeing a professional doctor. He went to the doctor only once, but it stopped	NO service usage
3	1	F	80’s	Unspecified	CGSC was suddenly telephoned from the CGSC of the south pole of the county in which she was sheltered	Her brother traveled to there to bring her back	NO service usage
4	1	F	60’s	Unspecified	Her son contacted the CGSC when she moved to the area, saying that she was repeating hospitalization, and she suffered from extravagance	CGSC visited her and suggested seeing a professional doctor, which failed	NO service usage
5	1	M	70’s	Dementia	He stopped visiting doctors of memory impairment and some physical impairment. A welfare commissionaire brought him to CGSC	CGSC struggled because he could not control his anger, but he finally visited a doctor	Clinic
6	2	M	80’s	Suicide attempt	She telephoned CGSC that she failed to conduct a hanging	Staff of CGSC rushed to her house and arranged doctor visitation	Clinic
7	2	F	70’s	Suicide attempt	Her husband telephoned CGSC that she was beating herself with a glass bottle	Staff of CGSC rushed to her house and arranged visiting a doctor. However, she refused. CGSC then arranged for a doctor to visit her house, and it was effective	Clinic
8	2	F	70’s	Bipolar disorder	She picked up the flowers from the flower bed on the street. She also visited her friends without permission	CGSC suggested going to a day-care center for older people and succeeded	Community service (not mental health)
9	2	F	80’s	Schizophrenia	She stayed in a bus stop with an unclean appearance. She also talked with the ancient emperor	Staff brought her to a voluntary community café with the help of the neighbors, and they welcomed her	Community service (not mental health)
10	2	M	70’s	Developmental disability	Although his cognitive test remained normal, he fell for a fraud	CGSC suggested seeing a professional doctor and succeeded	Clinic
11	2	F + F(sisters)	unknown	Delusional disorder	Old sisters living by themselves. They had a delusion that some famous artist lived in their flat as they insisted they smelt paint thinner	Ongoing	Ongoing
12	3	M	70’s	Alcohol misuse	He continued drinking day and night and could not eat any more	Staff of CGSC visited his house every day for two months, and he was willing to go to hospital for care. He then moved to a care house	Hospitalized
13	3	M	80’s	Unspecified	He was beating his wife	Staff of CGSC visited his house and called the police. However, involuntary hospitalization was denied by the authorities. After a struggle, he was hospitalized on family agreement hospitalization	Hospitalized
14	3	F	80’s	Depression	She was depressed after her husband died	CGSC suggested seeing the professional doctor and succeeded	Clinic
15	3	F	70’s	Dementia	After her husband’s death, she was living with her son. She had schizophrenia and her son had undiagnosed intellectual disabilities	CGSC suggested to going to a day-care center for the older people and succeeded	Community service (not mental health)
16	3	M	40’s	Developmental disability	He was the son in the case mentioned above. He graduated from college. However he had limited intention to live	The problem was that there was no appropriate service for this case	NO service usage
17	3	M	70’s	Intermittent explosive disorder	He lived with his daughter who suffered from a personality disorder. Their relationship broke up	He was finally moved to a shelter for victims of domestic violence	NO service usage
18	4	M	50’s	Borderline intellectual disability	He was withdrawn and his father always shouted at him	The consultant doctor advised that forcing him to work would not work and creating a secure place for him was important	Community service (not mental health)
19	4	M	40’s	Depression	He was withdrawn and preached to by the doctors at the clinics	Ongoing	Ongoing
20	5	M	40’s	Substance abuse	He is currently in the criminal justice system. The social worker thought that he has ADHD, but he had never been diagnosed	The social workers prepared some place after he came out of jail, but they also thought that he might run away	Community service (not mental health)
21	5	M	40’s	Schizophrenia	When there was a stressor, he stole	Because his wife also has mental illness, his wife could not be calmed down	Community service (not mental health)
22	5	M	60’s	Unspecified	He was jailed repeatedly	Because he had no formal diagnosis, he could not receive enough support	NO service usage
23	6	F	50’s	Unspecified	She asked for support. However she hid her information and never allowed supporters to come into her house	The community workers did not find anything to do. In addition, she complained to the authorities	NO service usage
24	6	F	40’s	Unspecified	When she moved to the area, community supporters did not share information with each other	The social worker held a community care conference to share information. She recovered	Community service (not mental health)
25	7	M	40’s	Schizophrenia	He moved to the shelter from the street. He was difficult to communicate with	Staff suggested seeing a professional doctor and he got better	Clinic
26	7	M	40’s	Schizophrenia	He spoke of hearing hallucinations at midnight	Staff suggested seeing a professional doctor and he left the shelter	NO service usage
27	8	M	60’s	Schizophrenia	He was suspected to have cerebral infarction by the primary care physician. However, the brain images did not show any abnormalities	The social worker consulted a psychiatrist and he was found to have neuroleptic malignant syndrome	Hospitalized
28	8	F	60’s	Schizophrenia	She lived with her daughter who had schizophrenia	Ongoing	Ongoing
29	8	F	70’s	Suicide attempt	In the ward, she attempted suicide. When the nurses stopped her, she became violent and waved her cane	Her family refused to be engaged, and she was finally discharged from the hospital	NO service usage
30	8	F	80’s	Unspecified	She called a medical ambulance three times a day	The social worker shared the information with the CGSC	Community service (not mental health)
31	9	M	50’s	Dementia (Frontotemporal dementia)	His family was burned out by caring for his perioral symptoms	After he was treated in hospital, his symptoms reduced	Hospitalized
32	9	F	80’s	Self-neglect	Because she was dependent on her husband, she stopped caring for herself after her husband’s death	The social worker continued visiting her house. However, she died due to a physical condition	NO service usage

**Fig. 3:**
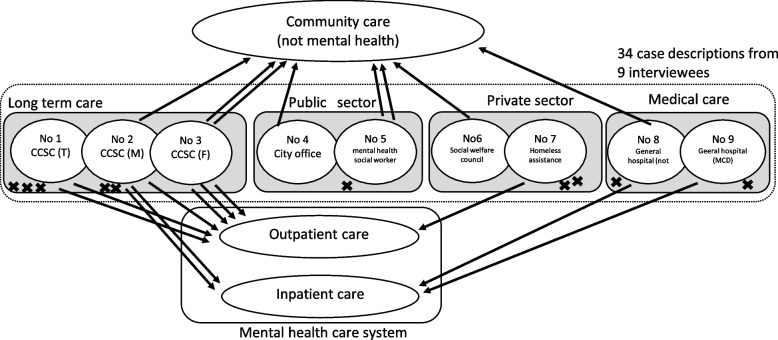
Overview of the outcomes of the cases involved in this study. *Note:* × stands for the outcome that resulted in no service usage. 11 cases were moved into the mental health care system (downward arrows), seven cases involved outpatient care, and four cases involved inpatient care; eight cases involved social care (upward arrows); 10 cases involved no service usage ( ×); and the remaining three were ongoing cases

Figure 3 illustrates the outcomes of the cases. A total of 11 cases moved into the mental health care system: seven in outpatient clinics and four hospitalized. Eight cases moved into community care, outside the mental health care system. Furthermore, 10 cases were referred to no service usage, and three were ongoing.

### Factors related to the non-utilization of formal mental health care systems

Concerning the factors related to the non-utilization of formal mental health care systems for the older people with urgent, unaware, and unmet mental health care needs, despite the existence of UHC, two over-arching categories emerged: “factors related to an individual person” and “factors related to the systems.” The categories included “individual intrinsic factors” and “family factors” for the former, and “physical health system related factors,” “mental health system-related factors,” “social service system related-factors,” and the “lack of integration between systems” for the latter. Some themes, such as “community people-related factors” and “factors related to inter-regional movement,” were not considered over-arching factors. The arrangements of the over-arching categories, factors, and sub-factors are listed in Table 3.

**Table 3:** Arrangements of the over-arching categories, factors, and sub-factors

Factors		Suggested solutions
Factors related to specific individuals		
Individual’s intrinsic factor	Difficulty in seeking help	Recognition of how older people may inadequately understand their health or be unaware of available services
	Delusions	Building a therapeutic alliance
Family factor	Discord between family members	Provision of particularly intensive care for families with family discord
	Denial of engagement	To have intensive efforts at contact and early involvement after contact with health care services
	Multiple cases in one family	Provision of particularly intensive care for families with multiple cases
	Family’s difficulty in seeking help	Provision of particularly intensive care for families who face difficulties in seeking help
Factors related to systems		
Physical health system-related factors	Indifference of physical healthcare providers regarding mental health matters	Mental health education campaigns to enhance mental health knowledge among non-mental health professionals
	Discontinuation of physical health conditions	
Mental health system-related factors	Irresponsive mental health care systems	Reforms to be more responsible, especially regarding emergency inpatient care
	Uncomfortable experiences in previous visits to clinics	Promote listening without ageism in clinical practice
Social service system-related factors	Lack of time to provide care	Expansion of social services regarding human resources, flexibility of use, and breadth of types of care provided
	Social service was not allowed without diagnosis	
	No appropriate service in the community	
Lack of integration between systems		Facilitate integration between the systems
Others		
Community people-related factors		Encouragement of the community residents to be involved in social security systems
Factors related to inter-regional movements		Provision of particularly intensive care for people who have just moved in

#### Factors related to a specific individual (over-arching category)

##### Individual intrinsic factors

Some cases involved the difficulty in receiving support from others, and as a result, they were named “difficulty in seeking help.”

*She has been working for some time, but she never disclosed such details to the supporters. Because she never let the supporters in her house, nobody could support her throughout her daily life. The supporters had to communicate with her on a park bench. She then filed a complaint with the administrative grievance office, saying that she was repeatedly told to let the supporters into her home (Interviewee No 6; on case 23).*

Cases involving “delusions” were expressed as significantly difficult cases by several interviewees, as follows:

*We have many cases involving delusions, and all of them are not connected to medical systems (Interviewee No 2; not on a specific case).*

*Individuals with delusions are difficult to support. In cases where hospitalization is driven by complaining neighbors, it is far more difficult to provide support for such individuals. Such patients no longer have delusions after hospitalization because of the change in environment. The sources of the delusions cannot be determined in the hospital environment. However, soon after discharge from the hospital, the delusions reappear (Interviewee No 9; not on a specific case) [Explanatory note: neighbors do not have the power to force people to get admitted to a hospital. However, they might have an influence on the proxy].*

##### Family factor

“Discord between family members” was reported to be a crucial factor.

*The client’s family who live together and the client’s parents who live in a different household were not aligned at all (Interviewee No 9; on case 31).*

In cases where the family denied engagement, the case work sometimes stopped. This was named “denial to engage.”

*There is a family in the distant region. However, they said that they could not do anything in case the hospital staff tried to make contact (Interviewee No 8; on case 29).*

According to the interviewees, “multiple cases in one family” made the situation significantly complex.

*The mother has dementia, the older son has schizophrenia, and the younger son has undiagnosed intellectual disabilities. After the father died, everything went wrong (Interviewee No 3; on cases 15 and 16).*

In addition to the client’s difficulty in seeking help, as described above, the “family's difficulty in seeking help” was also discussed.

*It is difficult to provide support if the husband is attempting to deal with the wife’s problem alone, and if the husband is older but still working, it is difficult for him to entrust his wife to someone else (Interviewee No 1; not on a specific case).*

#### Factors related to the systems (over-arching category)

##### Physical health system-related factors

In the physical health setting, people providing professional care to individuals suffering from mental illnesses were not well-equipped. Staff members without enough skills or knowledge were regarded as those in the “indifference of the physical health care provider regarding mental health matters” category.

*In general hospitals, people suffering from mental illnesses are sometimes regarded as troublemakers and are talked about in the contexts of the ways in which to quickly discharge them (Interviewee No 8*; *not on a specific case).*

Some people were out of the medical health care system, and they were placed in the category of “discontinuation of physical health conditions.”

*The problem was that he had infections resulting from the human immunodeficiency virus and the hepatitis B virus. However, he stopped seeing doctors (No 7; on one episode).*

#### Mental health system-related factors

Furthermore, the issue of “irresponsive mental health care systems” was also discussed.

*A person who was just discharged from a psychiatric hospital got worse again. When we asked the hospital about further inpatient care, they said that they cannot apply to the health insurance provider for the payment of additional acute care within three months after the previous discharge (Interviewee No 3; not on a specific case) [Explanatory note: In the end, the patient was accepted by the hospital].*

*(Despite the imminent violence against his wife and the destruction of his television using a hammer, after the arrival of the police, he was not admitted to the psychiatric emergency system by the authority because they said no one was injured during the incident　[Interviewee No 3; on case 13]).*

Some clients had “uncomfortable experiences in their previous visits to clinics.”

*We once went to a psychiatric clinic together, but he did not get along with the doctor, and he fell into a state of medical mistrust, saying that he would never go back (Interviewee No 7; on case 26).*

### Social service system-related factors

Some of the staff members did not have enough time to support people suffering from mental health challenges, and this category was named as “lack of time to provide care.”

*In our work settings, we cannot repeat interviews like psychologists. If we communicate intensively for a long time, the patients might start talking about previous traumatic experiences (Interviewee No 1; not on a specific case).*

The limitations of social welfare systems were also discussed, especially as they pertained to cases without rigid diagnosis, and this category was named “social service was not allowed without diagnosis.”

*In short, if one is not eligible for drug therapy, clinics will not readily accept them (Interviewee No 4).*

#### You cannot use social welfare support because you do not have a disability (Interviewee No 5).

Sometimes they found “no appropriate service in the community.”

*Concerning individuals with developmental disabilities, medical staff remained negative. Public health nurses said that they cannot deal with such individuals. The staff of the center for developmental disorders said that the services can commence only after the person comes to the center (Interviewee No 3; on case 16).*

### Lack of integration between the systems

The lack of integration between the systems was repeatedly mentioned.

*They say, “We can only help you so far.” With an increase in the number of various systems, the subject falls between the cracks (Interviewee No 3; not on a specific case).*

*We should work based on the idea of the ways in which we can solve the problems in front of us, and we should not work within the job description given to us (Interviewee No 5; not on a specific case).*

*There are many different parties involved, but each party is working on a single problem of the client. Therefore, information is not being shared between the parties involved (Interviewee No 6; not on a specific case).*

#### Others (outside the over-arching categories)

##### Community people-related factors

In addition to the individual and system factors, community people played a significant role in ensuring social inclusion.

*Until then, people in the community had been helping them in various ways. However, with the introduction of long-term care insurance services, local people are becoming increasingly reserved or at ease, and as a result, they are no longer involved. Until then, people in the community helped such individuals, but currently, they find themselves in situations where the only people around them are professionals (Interviewee No 3; not on a specific case).*

##### Factors related to inter-regional movement

As per clinical knowledge, it was reported that the moment of just having moved to another area was a crucial moment.

*As for those who have recently moved to the area, the names of their previous residences are personal information, and we do not have any knowledge of such information. We can only ask the patient. We are not allowed to collect information about such individuals unless they are a nuisance or otherwise, significantly serious. Therefore, we do not know anything about someone who has just moved into the area (Interviewee No 1; not on a specific case).*

## Discussion

This study explored the factors related to the non-utilization of formal mental health care systems among middle-aged and older people with urgent, unaware, and unmet mental health care needs, despite the existence of UHC. As per the results, two over-arching categories emerged, i.e., “factors related to an individual” and “factors related to systems,” which suggested certain targets for intervention, as discussed below. In addition to the factors under these categories, “inter-regional movement” and “community people-related factors” also emerged in this study.

Individual intrinsic factors involved the “difficulty in seeking help” and “delusional disorders,” consistent with previous literature as well as our clinical experiences. Concerning the difficulty in seeking help, the low rates of professional mental health service usage among older people has been reported in previous studies [27]. Whether this underuse results from the negative attitudes toward seeking help remains controversial. Mackenzie et al. [28] reported that 80% of older people showed positive attitudes towards seeking help, and they concluded that providing resources, such as the access to properly trained geriatric mental health professionals, was necessary. From Japan, Murata reported that the realistic factors related to delayed care were cost, distance, transportation problems, and conditions not serious enough to require seeking help [29]. The recognition of the ways in which older people may inadequately understand their health or be unaware of available services has also been suggested [30]. Concerning another factor, i.e., delusional disorders, according to Lapid and Ho [31], older people suffering from delusional disorders did not seek help and were often undiagnosed and untreated. From a conventional view, little can be done for people suffering from delusional disorders. However, Nagendra et al. [32] reported a high recovery rate of delusional disorders among older people. This suggests that the initiation of therapy through mental health competence, such as building a therapeutic alliance, is the key to addressing such issues. Recently, a Japanese group reported a successful case involving an elderly woman with lifetime undiagnosed delusion. By welcoming her to the community space and not referring her immediately, and by taking enough time to build trust with each other, she eventually saw a psychiatrist and very little medication significantly improved her general health [33].

The family factor, specifically, that involving “multiple cases in one family,” was a phenomenon sometimes faced by the clinicians. In biological psychiatry, a multiplex family has been a major concern because it is the key to establishing the crucial gene behind the disease. As an extension of this context, the burden of care was reported to be highly significant for families with more than one ill member (multiplex) compared to families with a single affected individual (simplex) [34]. Contrarily, what we encountered in this study was not associated with genetic multiplexity. One family comprised individuals suffering from dementia, schizophrenia, and developmental disorders, whereas another comprised a married couple suffering from schizophrenia and ADHD. The results of this study suggested that the provision of particularly intensive care for families with multiple cases was inherent. The “denial for engagement” factor was also reported by Sung-Wan Kim et al. [35]. They suggested ensuring intensive efforts and early involvement after contact with health care services. As far as we searched, although it is evident that “discord between family members” affects the treatment of mental health problems form the clinical perspective, the evidence provided in this study is not enough. In the field of palliative care, family discord is reported to relate to stronger preferences for life-prolonging treatments and weaker preferences for palliative care [36]. Concerning the “family’s attitude toward seeking help,” the associated literature is also sparse. Mc Gonagh explored the factor of family’s attitude among adolescents suffering from anxiety disorders, and they reported that the family’s attitude affected the patients’ attitudes [37]. These findings suggest that this pattern may be similar for family relationships among older people with mental health care needs.

This study suggests the improvement of both the physical and mental health systems. In this study, concerning physical health systems, the indifference of medical health staff regarding the provision of care to people with mental health care needs emerged. Wu et al. [38] conducted a mental health literacy survey involving non-mental health professionals in general hospitals throughout China, and they established that the beliefs that non-mental health professionals held regarding mental disorders were inadequate for ensuring the provision of appropriate help. Additionally, previous studies show that people with psychosis have low levels of physical health literacy [39]. There are challenges for both providers and users, as they pertain to the appropriate delivery of physical health care to people with mental health care needs. Wu et al. [38] suggested the implementation of mental health education campaigns to enhance mental health knowledge among non-mental health professionals.

Concerning mental health care systems, the “irresponsive mental health care system” factor was merged in this study. Mental health care systems should ensure increased responsibility, especially regarding emergency inpatient care. Regarding the provision of social services, the lack of human resources, the excessive need for medical diagnoses, and the narrowness of the prepared services were discussed in this study. In this study, we advocated for the expansion of social services, as they pertain to human resources, the flexibility of use, and the breadth of the types of care provided. Additionally, “uncomfortable experiences in previous visits to clinicians” were noted in this study. Previous studies show that older people experience being treated differently as a result of ageism [40] or not being listened to fully from the beginning of the treatment of their mental illnesses [41]. A new finding, “just moved to a new region,” was a decisive factor. Community ties continue to weaken, especially in urban areas. As long as informed consent was obtained, visits by mental health professionals to people who had just moved to the region was one method for strengthening social support networks among communities and including older people at the potential risk of mental disorders.

In this study, we also suggested that social service systems are not responsive to older people with mental health care needs because 1) the staff do not have enough time, 2) the staff cannot respond freely without diagnoses, 3) and no appropriate service menus are prepared when the older people with urgent, unaware, and unmet mental health care needs contact social services. To the best of our knowledge, there are no studies investigating this issue. From the limited data obtained in this study, we suggest the expansion of social services, as they pertain to human resources, the flexibility of use, and the breadth of the types of care provided.

Concerning the “lack of integration between the systems,” the Ministry of Health, Labor, and Welfare of Japan points out that vertically segmented administrative systems present a point of weakness in Japan’s social security system [42]. This is an institutional problem that is too large to be the subject of research, and no empirical studies pertinent to clinical practice could be found. However, the phenomenon of older people with urgent, unaware, and unmet mental health care needs remains a satisfactory guide for future institutional design in that it clearly demonstrates the existence of this problem. In this study, the “community people-related factor” specifically involved the lack of continuity in the involvement of care among community residents. This is another way of stating the lack of integration between various social security systems and the communities involved.

No overt spiritual needs were discussed in this study. However, this was because we only interviewed professionals and not individuals. Therefore, it is natural that the objectively assessed medical and social needs were discussed first. In our previous study, we reported the possibility that medical professionals disclosed their suffering only in the presence of a priest [43].

## Conclusions

In this study, the factors related to the non-utilization of formal mental health care systems among middle-aged and older people with urgent, unaware, and unmet mental health care needs, despite the existence of UHC, were categorized, and they suggested the requirement of a specific target for intervention. The interventions discussed in this study are listed in Table 3. Concerning the individual intrinsic factors, for the factor associated with difficulty in seeking help, we suggested the public and professional recognition of the ways in which older people may inadequately understand their health or be unaware of the services available. Regarding the factor of delusions, we suggested the formulation and implementation of a therapeutic alliance. Regarding the family factor, we suggested the provision of particularly intensive care for families with family discord, families with multiple cases, and families facing difficulties in seeking help. To facilitate family engagement, intensive efforts and early involvement after achieving contact with mental health care services are necessary. Concerning the physical health system-related factor, mental health education campaigns aimed at enhancing mental health knowledge among non-mental health professionals are essential. For the mental health system-related factor, reforms aimed at increasing responsibility levels, especially regarding emergency inpatient care, and promoting listening without ageism in clinical practice are essential. For the social service system-related factor, the expansion of social services regarding human resources, the flexibility of use, and the breadth of the types of care provided are required. Regarding the lack of integration between the systems, facilitating the integration between the systems is necessary, which is Japan’s policy goal, as described above. Regarding the community people-related factor, encouraging community residents to involve themselves in social security systems is necessary, and finally, for the factors related to inter-regional movement, the provision of particularly intensive care for people who have just moved to new areas might be promising. Among such factors, novel and realistic insights explaining the reasons why inter-regional movements are critical among various individuals can be achieved. Accordingly, the interventions provided to older people at the time of movement can be considered variables in future studies on this subject.

### Limitations and directions for future research

This study has several limitations. First, the interviewees were selected through snowball sampling, with inclusion criteria. As a result, the interviewees’ representativeness was not robust. Second, we suggested an interdisciplinary interviewer team’s function for unspoken views. However, we cannot compare this with control interviews, such as interviews conducted by psychiatrists alone among the same interviewees. Third, this study comprised interviews involving experts or care providers. The advantage of this study was that the facts were organized, and the viewpoints were fixed. However, there had to be facts and views from the care recipients’ side, which were not discussed in the supporters’ interviews. Interviews involving the latter are required to explore the inner need of individuals and understand the unbiased views of both the supporter and the individual. However, the access to such individuals was not possible after they became people with urgent, unaware, and unmet mental health care needs. In this study, we revealed that inter-regional movement was a critical point. Accordingly, people who moved were candidates of future studies. Therefore, it is feasible to broadly reach out to people in our catchment area who have just moved into the community, build relationships, and investigate the internal struggles of those with mental health needs.

## Data Availability

The datasets generated and/or analyzed during this study are not publicly available owing to the protection of privacy. This study represents qualitative research by conducting interviews among practitioners from the authors’ professional network. It is not possible to publish the interview transcripts. Even in anonymized transcripts, it is not possible to identify specific participants owing to their personal connections to the authors. However, the datasets used in this study are available from the corresponding author upon reasonable request.

## Abbreviations

UHC: Universal Health Coverage
CGSC: Community General Support Centers
MCD: Medical Centers for Dementia
NPO: Non-Profit Organization
